# Meta-analyses of the effects of high-intensity interval training in elite athletes — part II: relationships between the mean effects on various performance measures

**DOI:** 10.3389/fphys.2024.1486570

**Published:** 2024-12-23

**Authors:** Hans-Peter Wiesinger, Will G. Hopkins, Nils Haller, Julia Blumkaitis, Tilmann Strepp, Thomas Leonhard Stöggl

**Affiliations:** ^1^ Department of Sport and Exercise Science, Paris Lodron University Salzburg, Salzburg, Austria; ^2^ Institute of Nursing Science and Practice, Center for Public Health and Healthcare Research, Paracelsus Medical University, Salzburg, Austria; ^3^ Institute of General Practice, Family Medicine and Preventive Medicine, Center for Public Health and Healthcare Research, Paracelsus Medical University, Salzburg, Austria; ^4^ Internet Society for Sport Science, Auckland, New Zealand; ^5^ Department of Sports Medicine, Rehabilitation and Disease Prevention, Johannes Gutenberg University, Mainz, Germany; ^6^ Red Bull Athlete Performance Center, Thalgau, Austria

**Keywords:** meta-analyses, endurance, team sports, elite athletes, mediation

## Abstract

**Introduction:**

Our recent meta-analyses have demonstrated that high-intensity interval training (HIIT) causes a range of mean changes in various measures and predictors of endurance and sprint performance in athletes. Here, we extend the analyses to relationships between mean changes of these measures and consider implications for understanding and improving HIIT that were not apparent in the previous analyses.

**Methods:**

The data were mean changes from HIIT with highly trained endurance and elite other (mainly team sport) athletes in studies where two or more measures or predictors of performance were available. Relationships between changes in pairs of measures were visualized in scatterplots with points identified by aerobic and anaerobic types of HIIT; simple linear relationships were quantified via log-transformation of factor changes with a meta-regression mixed model.

**Results:**

In endurance athletes, there were positive linear relationships between mean changes in time-trial speed/power (reflecting competition endurance performance) and mean changes in endurance performance predictors [peak speed/power, maximal oxygen uptake (V̇O_2max_), and aerobic/anaerobic threshold]. There were substantial differences in time-trial speed/power between studies not explained by each predictor. Exercise economy had an unclear relationship with time-trial speed/power but a decisively negative relationship with V̇O_2max_. In other athletes, repeated-sprint ability had a weak positive relationship with sprint speed/power. The scatter of points in some plots was associated with the type of HIIT.

**Discussion:**

Differences in time-trial performance between studies for a given change in peak speed/power, V̇O_2max_, or threshold speed/power imply that time trials should be included when assessing effects of HIIT on endurance performance. Relationships between V̇O_2max_, time-trial speed/power, and exercise economy suggest that combining aerobic and anaerobic types of HIIT could be more effective for endurance performance. Sprints and repeated-sprint ability are important performance measures for team-sport athletes; their poor relationship implies that both should be measured when assessing HIIT.

**Clinical Trial Registration:**

https://www.crd.york.ac.uk/PROSPERO/display_record.php?RecordID=236384

## 1 Introduction

In our recent meta-analyses (Wiesinger et al., 2024), we found that high-intensity interval training (HIIT) caused substantial enhancements in time-trial, repeated-sprint, and sprint performance of highly trained athletes in many study settings. In addition to these measures of performance, HIIT improved various predictors of endurance performance: V̇O_2max_, peak speed or power, exercise economy, and aerobic/anaerobic threshold speed or power. However, we meta-analyzed the effects of HIIT on each measure and each predictor of performance separately. Here, we have investigated the relationships between the HIIT-induced changes in measures of performance and performance predictors.

There was a limited number of study estimates available for each analysis of these relationships. We have therefore opted to quantify the relationships via meta-regressions that included only one predictor of performance. Including two or more predictors would reduce the number of study estimates even further, resulting in unacceptable uncertainty in the relationships. For the same reason, we have excluded from quantitative analyses any moderators of HIIT, but we have incorporated some moderators visually in plots of relationships between the variables. The aim of these meta-analyses was to understand the effects of HIIT by quantifying the magnitude and uncertainty in the relationships between measures and predictors of performance and consequently to discuss the implications for improving HIIT.

## 2 Methods

To meta-analyze the relationships between mean changes in different types of HIIT and control training on performance-related measures, we drew upon 23 studies in the companion paper (Wiesinger et al., 2024) containing at least two measures or predictors of performance: here, 12 studies were conducted on highly trained classical endurance athletes [runners (Smith et al., 2003; Stöggl and Sperlich, 2014; Salazar-Martinez et al., 2018), cyclists (Stöggl and Sperlich, 2014; Salazar-Martinez et al., 2018; Clark et al., 2014; Skovereng et al., 2018; Rønnestad and Vikmoen, 2019; Sylta et al., 2016; Laursen et al., 2002; Stepto et al., 1999), duathletes or triathletes (Smith et al., 2003; Stöggl and Sperlich, 2014; Salazar-Martinez et al., 2018; Laursen et al., 2002), cross-country skiers (Stöggl and Sperlich, 2014; Sandbakk et al., 2013; Sandbakk et al., 2011), and rowers (Stevens et al., 2015)]; the remaining 11 studies included elite other athletes [first league, national team, or international level in ball sports (Wells et al., 2014; Helgerud et al., 2001; Akdoğan et al., 2021; Iaia et al., 2015; Soares-Caldeira et al., 2014; Thomassen et al., 2010; Hermassi et al., 2018; Selmi et al., 2018), canoe sports (Sheykhlouvand et al., 2018; Yang et al., 2017), and alpine skiers (Breil et al., 2010)]. The PRISMA flow diagram and the inclusion criteria for these studies were comprehensively outlined in Part I (Wiesinger et al., 2024). In our other recent paper (Stöggl et al., 2024), six different types of HIIT were justified: Type 1 (most aerobic) through to Type 6 (most anaerobic). These are color-coded in the scatterplots of the present paper to facilitate discussion of potential modifying effects of HIIT. Note that no studies with the most anaerobic type of HIIT had at least two measures or predictors of performance. Therefore, conclusions about sprint interval training (SIT) cannot be drawn from the current analysis without extrapolating beyond the data.

The data were the mean changes and their standard errors for each study-estimate (expressed as percent) of each outcome measure in the previous study (Wiesinger et al., 2024). The percent mean changes and standard errors were expressed as factors and log-transformed for meta-regression analyses between all pairs of measures with sufficient changes separately for HIIT and control data. The residual variance was set to unity to perform the weighting via the inverse square of the standard error of the dependent mean-change variable (Rønnestad and Vikmoen, 2019; Yang, 2003). The fixed effects were the linear-numeric mean-change predictor variable and the intercept. The random effect was sample-estimate identity, a measure of heterogeneity that, in this model, represents prediction error when estimated as a standard deviation. The coefficient of the predictor (the slope) was interpreted directly as the percent change in the dependent variable associated with a 1% change in the predictor variable. A factor representing attenuation of the slope arising from uncertainty in the predictor (Hopkins et al., 2009) was calculated as the intraclass-correlation coefficient, given approximately by (SD^2^−SE^2^)/SD^2^, where SD is the observed standard deviation of the predictor and SE^2^ is the mean of the square of the standard errors of each value of the predictor. The slope and its confidence limits were corrected for attenuation by dividing by the factor. The mean value of the dependent variable was estimated with the same mixed model but omitting the predictor variable, and the corresponding mean value of the predictor was estimated from the slope and intercept of the first model. We excluded time trials <75 s [studies (Sylta et al., 2016; Stevens et al., 2015)] from the estimation of all statistics when time-trial speed/power was the dependent variable because the predictor variables were all measures of aerobic performance and were not expected to predict performance in such short time trials.

## 3 Results

**FIGURE 1:**
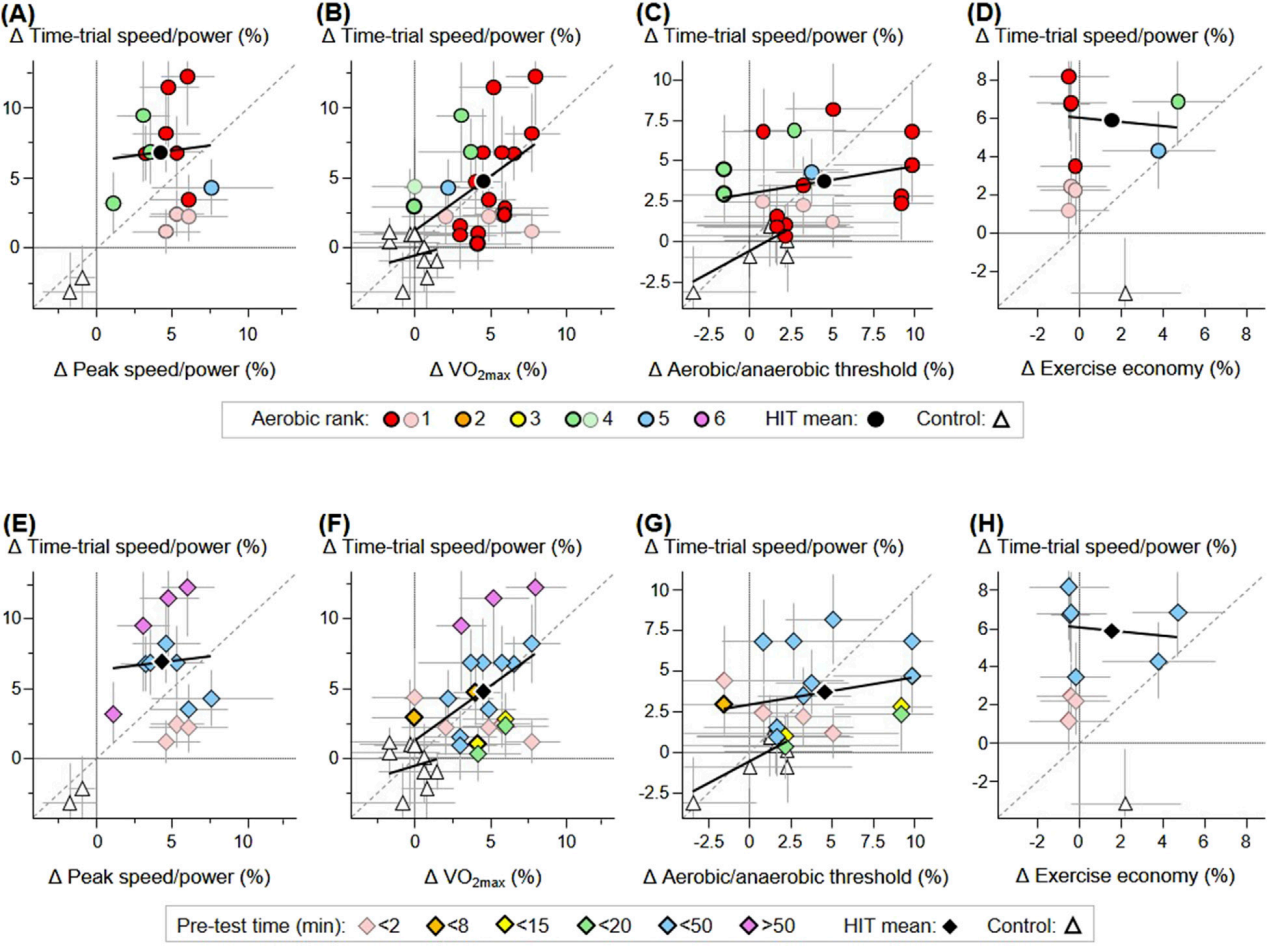
Scatterplots of study-estimate mean change scores (Δ) with time-trial speed/power as the dependent variable and the other outcome measures as the predictors. **(A–D)** show HIIT change scores identified with the type of HIIT, while **(E–H)** show the same data identified with pre-test time-trial duration. Regression lines are shown where there were sufficient HIIT or control data. Grand mean values are shown only for HIIT data and were derived via the weighting by the inverse of the standard error squared. The dashed gray lines are lines of identity. Pale symbols are study estimates for pre-test time-trial duration <75 s, which were excluded from the regression analyses. Error bars are 90% confidence intervals, some of which are truncated at the figure boundaries. Types of HIIT (Stöggl et al., 2024) were traditional long intervals (Type 1), intermittent short intervals (Type 2), speed endurance maintenance training (Type 3), speed endurance production training (Type 4), repeated sprint interval training (Type 5), and sprint interval training (Type 6).

**FIGURE 2:**
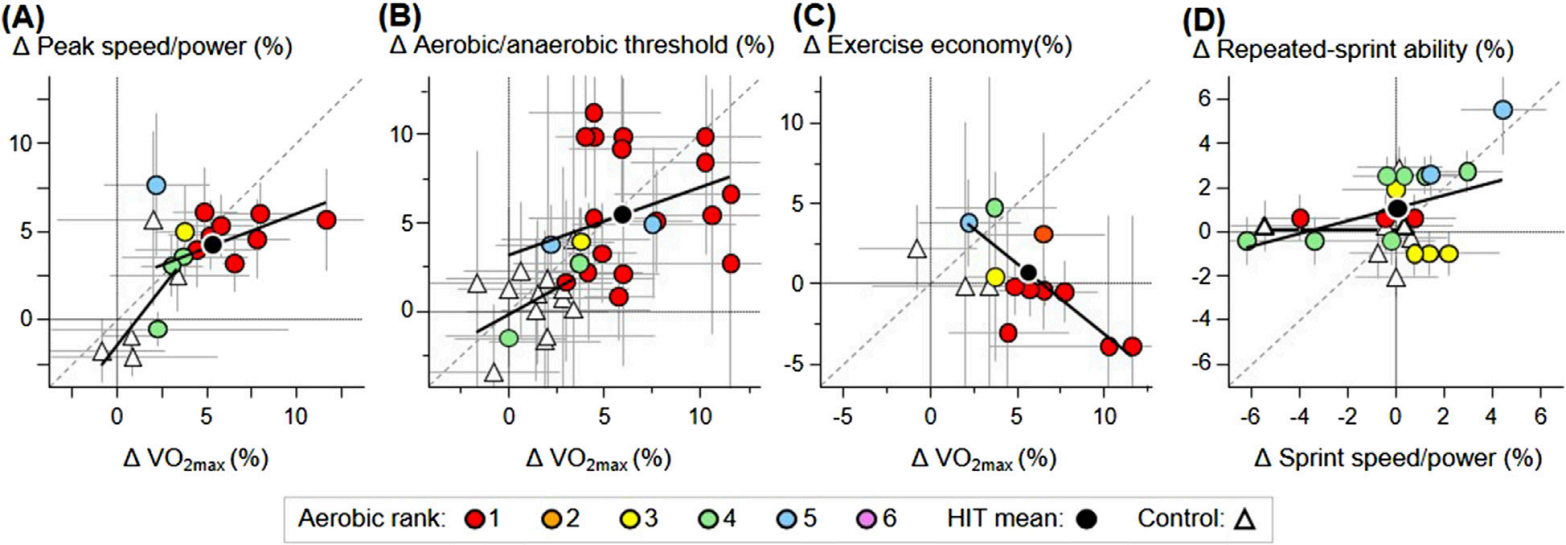
Scatterplots of study-estimate mean change scores (Δ) with pairs of dependent and predictor variables not shown in Figure 1. **(A-D)** HIIT change scores are identified with the type of HIIT. Regression lines are shown where there were sufficient HIIT or control data. Grand mean values are shown only for HIIT data and were derived via the weighting by the inverse of the standard error squared. The dashed gray lines are lines of identity. Error bars are 90% confidence intervals, some of which are truncated at the figure boundaries. Types of HIIT (Stöggl et al., 2024) were traditional long intervals (Type 1), intermittent short intervals (Type 2), speed endurance maintenance training (Type 3), speed endurance production training (Type 4), repeated-sprint interval training (Type 5), and sprint interval training (Type 6).

The mean changes in performance and performance predictors from each study are shown in a Supplementary Table. Figures 1, 2 show scatterplots for pairs of variables where there were sufficient estimates to allow analyses of the relationships. Figure 1 shows scatterplots of mean changes in time-trial speed/power and mean changes in performance predictors for elite endurance athletes, color-coded by the type of HIIT (A-D) and time-trial duration (E-H). Figure 2 shows scatterplots of mean changes between the performance predictors, predominantly for the highly trained endurance athletes, and between the mean changes in sprint speed/power and repeated-sprint ability for the elite other athletes.

Positive linear trends are apparent between time-trial speed/power as the dependent variable and peak speed/power, V̇O_2max_, and aerobic/anaerobic threshold as predictors (Figures 1A–C). Trends amongst these three predictors are also positive (Figures 2A, B), as is the trend between sprint speed/power and repeated-sprint ability (Figure 2D). Exercise economy shows little apparent relationship with time-trial speed/power (Figure 1D), and there is a negative relationship between exercise economy and V̇O_2max_ (Figure 2C). The pale symbols in Figure 1, representing estimates for pre-test time-trial duration <75 s, have an insufficient range of values for the predictor peak speed/power (Figure 1A) and exercise economy (Figure 1D) to allow interpretation of the relationships with time-trial speed/power; however, there is a tendency for negative slopes with V̇O_2max_ (Figure 1B) and aerobic/anaerobic threshold (Figure 1C).

There are barely discernable associations with the type of HIIT shown in Figure 2C (the greatest gains in aerobic/anaerobic threshold and V̇O_2max_ for the most aerobic HIIT) and Figure 2C (the greatest gains in exercise economy and least gains in V̇O_2max_ for the more anaerobic types of HIIT). There are no discernable associations in the other figures, but the types of HIIT for some study estimates are noteworthy. Specifically, in Figure 1D, there are substantial increases in time-trial speed/power and exercise economy in two settings where the type of HIIT was more anaerobic, and in Figure 2D, the greatest gains in sprint speed/power and repeated-sprint ability were achieved in a study (Selmi et al., 2018) with the most anaerobic type of HIIT (Level 5 on the six-point scale), but the greatest negative effect on sprint speed/power occurred in a study with HIIT that was only one step less anaerobic (Level 4).

Where sufficient estimates from control groups allowed estimations of slopes, there is some evidence of positive relationships between time-trial speed/power as the dependent variable and the predictors V̇O_2max_, and the aerobic/anaerobic threshold (Figures 1B, C). There is evidence of positive relationships between peak speed/power or the aerobic/anaerobic threshold and the predictor V̇O_2max_ (Figures 2A, B). Although a slope could not be estimated from control groups between exercise economy and V̇O_2max_, there is a tendency for a negative slope (Figure 2C).

Table 1 summarizes the HIIT data in Figures 1, 2 as grand means for each variable as the slopes of the linear relationships between the dependent and predictor variables and as the prediction errors. The grand means for time-trial speed/power tended to be greater than those for the performance predictors, especially for peak speed/power and exercise economy. Linear predictions of time-trial speed/power were characterized by moderate prediction errors, and slopes with sufficient uncertainty were observed for the population slopes to be negative or practically zero at the lower confidence and greater than 1%/% at the upper limits for peak speed/power and V̇O_2max_. After adjustment for uncertainty in the predictor, the confidence intervals of the slopes for peak speed/power and V̇O_2max_ included 1%/%, while those for the aerobic/anaerobic threshold and exercise economy were less than 1%/%.

**TABLE 1:** Effects of HIIT across studies providing means of each of two outcome measures, and the linear relationship between the means expressed as the prediction error, observed slope, and adjusted slope between dependent and predictor variables.

Dependent vs. predictor	Grand mean ± SD^a^ (%)		Prediction error^b^ (%)	Slope (%/%); ±90% CI	
	Dependent	Predictor		Observed^c^	Adjusted^d^
Time-trial speed/power vs. peak speed/power	7.6 ± 3.4	4.7 ± 1.9	2.6	0.14; ±1.03	0.23; ±1.24
Time-trial speed/power vs. V̇O_2max_	5.3 ± 3.7	4.7 ± 2.0	2.5	0.77; ±0.62	1.31; ±1.05
Time-trial speed/power vs. aerobic/anaerobic threshold	4.6 ± 3.9	3.9 ± 2.5	2.0	0.17; ±0.29	0.24; ±0.41
Time-trial speed/power vs. exercise economy	6.3 ± 1.9	1.2 ± 2.5	1.5	−0.11; ±0.77	−0.13; ±0.97
Peak speed/power vs. V̇O_2max_	4.4 ± 2.2	6.0 ± 3.3	1.5	0.40; ±0.41	0.63; ±0.64
Aerobic/anaerobic threshold vs. V̇O_2max_	5.3 ± 3.6	6.0 ± 3.2	2.3	0.38; ±0.41	0.51; ±0.55
Exercise economy vs. V̇O_2max_	0.0 ± 3.0	6.4 ± 3.1	0.7	−0.94; ±0.51	−1.29; ±0.70
Repeated-sprint ability vs. sprint speed/power	1.1 ± 1.9	0.1 ± 2.7	1.5	0.29; ±0.30	0.33; ±0.34

Data contributing to each row of the table are the undimmed points for the HIIT group in the corresponding plots of Figures 1, 2.

^a^ Simple statistics for the two outcome measures, not identical with the weighted grand means in the figures.

^b^ The scatter of points around the regression line.

^c^ The slopes are those of the regression lines for the HIIT group shown in the figures.

^d^ The slope corrected for attenuation arising from uncertainty in the predictor.

Among the predictors, grand mean changes for peak speed/power, the aerobic/anaerobic threshold, and especially exercise economy tended to be less than that for V̇O_2max_. Repeated-sprint ability tended to have greater grand means than sprint speed/power. Exercise economy had a small prediction error in its linear relationship with V̇O_2max_ and a slope close to −1%/% that was negative even at its upper confidence limit. The other relationships were characterized by moderate prediction errors and slopes of less than 1%/% at their upper confidence limits, although after adjustment, the confidence intervals included unity for peak speed/power and the anaerobic threshold predicted by V̇O_2max_.

## 4 Discussion

It is evident from the scatterplots and quantification of the linear relationships that none of the predictors of performance alone adequately characterizes the effect of HIIT on time-trial speed/power of highly trained athletes. Even for V̇O_2max_, where the observed slope with time-trial speed/power was closest to 1%/% and the mean changes were similar, the prediction error represents an impractical range of effects on performance in different settings. Anyone wishing to determine the mean effect of HIIT on their endurance athletes should, therefore, include an assessment of time-trial speed/power.

In the control groups, the small number of study estimates and the more limited range of changes resulted in too much uncertainty in the linear relationships to justify presenting them in Table 1. Although it is evident from the figures that the slopes in the control groups were similar to those in HIIT groups, a larger number of estimates in both groups might reveal differences in the slopes due to differences in the effects of HIIT and control training on endurance performance and its predictors (and on sprint speed/power or the repeated-sprint ability of other athletes).

The lack of an obvious linear association of the type of HIIT with the trends between time-trial speed/power and predictors is consistent with the finding in our previous study (Wiesinger et al., 2024). In that study, we observed that the type of HIIT had a reasonably well-defined moderating effect on V̇O_2max_ and aerobic/anaerobic threshold but not on time-trial speed/power and the other performance predictors. Nonetheless, sampling uncertainty allows that there could be a negative relationship across the board for endurance performance and predictors, indicating the need for more research. Furthermore, the need for further research becomes apparent as additional studies assessing several performance predictors would enable a multiple linear meta-regression. This approach, considering performance predictors in combination, would likely account for time-trial speed/power with better precision. With the improved prediction of time-trial speed/power in such analyses, the type of HIIT would likely also emerge as a substantial modifier, if it were included. Practitioners should nevertheless consider the measurement of several performance predictors to identify strengths and weaknesses in specific athletes that might allow for customization of HIIT or inclusion of other training interventions with individual athletes in a given setting.

To explore the relationships between endurance performance and its potential predictors, we excluded the shortest time trials, which, as expected, did not show substantial positive relationships in the scatterplots. With enough study estimates, it would be more appropriate to include all time trials in the analyses, with an interaction between the time-trial duration and the given predictor or predictors to properly account for the modifying effect of duration on the strength of the relationships. The precision of the estimates of the relationships would also be improved thereby. Such analyses might even reveal negative relationships between time-trial speed/power and its predictors for the shortest time trials, of which there were some suggestions with the present data. This outcome would arise if HIIT improved the aerobic performance at the expense of anaerobic performance contributing to these shortest time trials.

After adjustment, the bivariate relationships revealed the intriguing possibility that differences in the mean effect of HIIT on V̇O_2max_ were, on average, actually greater than the differences on time-trial speed/power (a slope greater than 1%/%) but were offset, to some extent, by impairments in exercise economy (a slope on the order of −1%/%). Understanding the mechanisms of these contrasting effects of aerobic or anaerobic types of HIIT on V̇O_2max_ and exercise economy, including the adverse effects of aerobic HIIT on exercise economy, is important for refining training interventions. One plausible avenue for further investigation is the biomechanical adaptations within the muscle–tendon unit. Elite athletes of different sports have distinctive tendon properties (Wiesinger et al., 2016; Wiesinger et al., 2017), with tendons of endurance athletes likely adapted to reduce the metabolic cost of muscle force production (Fletcher et al., 2013; Lichtwark and Wilson, 2007) and enhance the release of stored energy (Farris and Sawicki, 2012; Roberts, 2016). Although these athletes likely possess a well-developed exercise economy, there seems to be untapped potential for improvements. A strength intervention study on recreational runners explicitly designed to increase triceps surae tendon-aponeurosis stiffness (by ∼16%) substantially enhanced exercise economy (by ∼4%) (Alberts et al., 2012). Although achieving further improvements in exercise economy in highly trained athletes is likely more challenging, the hypothesis that anaerobic HIIT instead of strength training holds potential for relevant muscle-tendon adaptations deserves further investigation. The impairments of exercise economy that offset the improvements in V̇O_2max_ appear to have occurred with the most aerobic type of HIIT, and one plausible explanation is a shift in substrate utilization. Previous research has suggested that the ratio of substrate utilization may shift from carbohydrates to lipids after several weeks of training (McArdle et al., 2010). On average, fatty acid oxidation demands more oxygen compared to glucose oxidation due to the inherent characteristics of the metabolic pathways involved (McArdle et al., 2010; Friedlander et al., 1998). In addition, a higher V̇O_2max_ facilitates greater lipid oxidation and, consequently, a greater oxygen cost during sub-maximal exercise (Pate et al., 1992). Thus, such changes in substrate utilization at fixed speeds/power may impact exercise economy, if there is a shift from efficient substrates (e.g., carbohydrates) to less efficient ones (e.g., fatty acids). To take into account substrate utilization, exercise economy might be more accurately defined as caloric unit cost (kcal·kg^−1^·km^−1^) (Barnes and Kilding, 2015). It may be possible to avoid this potentially negative effect of aerobic HIIT on exercise economy by incorporating aerobic and anaerobic types of HIIT in some periodized manner aimed at synergistic effects on endurance performance.

In contrast to V̇O_2max_, the aerobic/anaerobic threshold predicted less than a 1%/% change in time-trial speed/power even after adjustment for uncertainty in the threshold measure at its upper confidence limit. This result was unexpected, considering that the aerobic/anaerobic threshold, as quantified, should be a good measure for tracking endurance performance. Indeed, the grand mean changes in time-trial speed/power and the aerobic/anaerobic threshold after adjustment were similar (∼5%, Figures 1C, G), so evidently, there is some additional random error in the change in threshold such that it under- or overestimates the change in endurance performance, depending on how it was measured in different settings. A review of previous studies of the relationships between endurance performance and the various measures of aerobic and anaerobic threshold might reveal which measure tracks performance most accurately.

For team-sport athletes, sprints and the repeated-sprint ability are both important performance measures (Girard et al., 2011; Bishop et al., 2011), and the scatterplots and derived regression statistics show that both should be measured when assessing HIIT. Although the mean effects on the repeated-sprint ability and sprint speed/power were only marginally small and practically zero, respectively, much larger gains in both measures were achieved in one study (Selmi et al., 2018), apparently associated with the most anaerobic type of HIIT. Unfortunately, there were no other noticeable differences in the characteristics of the training or athletes in this particular setting compared with those of the other studies. If this study is an exception for any reason, eliminating it would aggravate the poor association between these two performance measures. This weak relationship can be explained by the fact that sprint performance is primarily determined by anaerobic power, whereas repeated-sprint performance is determined by aerobic and anaerobic power (Girard et al., 2011; Bishop et al., 2011); HIIT-induced improvements in the aerobic system could, thus, produce greater gains in the repeated-sprint ability than in sprint speed/power. A trade-off between aerobic and anaerobic adaptations might even result in detrimental alterations in sprint and/or repeated-sprint performance, as evident in some instances in Figure 2D. Researchers need to investigate whether the most anaerobic types of HIIT, alone or in periodized combination with aerobic HIIT, avoid any such trade-off and instead enhance both types of performance.

## 5 Limitations

As noted in the previous meta-analysis, it is important to acknowledge that authors have not assessed the effects of HIIT on competition performance (Vandenbogaerde et al., 2012). However, it is reasonable to assume that the mean effects estimated from the laboratory data would also apply to compare performance, on average, and thus have not introduced a bias in the position and slope of the regression lines.

The current meta-analyses have shown several critical gaps in the literature regarding the relationships of various measures and predictors of sprint and endurance performance. First, there is a need for further research to allow multiple linear meta-regression, facilitating the integration of various performance predictors in the model. Second, a deficiency exists in studies analyzing the effect of HIIT on various measures and predictors, including both sprint and endurance performance measures. Third, a more comprehensive understanding of the effects of standard control training is required, necessitating additional studies and detailed information on the methodologies employed in such training. Another notable gap is the unresolved nature of the mechanisms underlying the effects of HIIT, although our meta-analyses have shown several intriguing aspects and suggestions for future studies.

## 6 Conclusion

The substantial variability observed in change in mean time-trial performance between study settings for a given change in peak speed/power, V̇O_2max_, or threshold speed/power implies that these three test measures would not accurately reflect the effects of HIIT on performance in endurance competitions. Time trials should therefore be implemented whenever possible to accurately assess the effect of HIIT. The three test measures, along with exercise economy, might nevertheless identify strengths and weaknesses to customize further implementation of HIIT or other training interventions of the individual endurance athletes in a given study setting. Sprints and repeated-sprint ability are important performance measures for team-sport athletes; their poor relationship implies that both should be measured when assessing HIIT. Finally, our meta-analyses provide evidence of substantial improvements in sprint and endurance performance of highly trained endurance athletes and elite other athletes, following existing types of HIIT, but combining aerobic and anaerobic types of HIIT may be more effective than the single types of HIIT employed in the meta-analyzed studies.

## Data Availability

The original contributions presented in the study are included in the article/Supplementary Material; further inquiries can be directed to hans-peter.wiesinger@pmu.ac.at.
