# Supplementary material for: Meta-analyses of the effects of high-intensity interval training in elite athletes — part II: relationships between the mean effects on various performance measures
Source: Front Physiol. 2024 Dec 23;15:1486570. doi: 10.3389/fphys.2024.1486570 (PMC11701006; doi:10.3389/fphys.2024.1486570)
Supplement: Supplementary file 1 [file Table1.docx]

**Title**

Meta-analyses of the effects of high-intensity interval training in elite athletes – Part II: relationships between the mean effects on various performance measures

**Supplementary appendix**

**This appendix formed part of the original submission**

**Running title**

Relationship of various performance measures

**Authors**

Hans-Peter Wiesinger,^a,b,c^ Will G. Hopkins,^d^ Nils Haller,^a,e^ Julia Blumkaitis,^a^ Tilmann Strepp,^a^ Thomas L. Stöggl^af^

^a^ Department of Sport and Exercise Science, Paris Lodron University Salzburg, 5400 Hallein-Rif, Austria.

^b^ Institute of Nursing Science and Practice, Center for Public Health and Healthcare Research, Paracelsus Medical University, 5020 Salzburg, Austria

^c^ Institute of General Practice, Family Medicine and Preventive Medicine, Center for Public Health and Healthcare Research, Paracelsus Medical University, 5020 Salzburg, Austria

^d^ Red Bull Athlete Performance Center, 5303 Thalgau, Austria

^e^ Department of Sports Medicine, Rehabilitation and Disease Prevention, Johannes Gutenberg University, Mainz 55122, Germany

^f^ Internet Society for Sport Science, Auckland, New Zealand

*Corresponding author:

Nils Haller

Department of Sport and Exercise Science, Paris Lodron University Salzburg

Schlossallee 49

5400 Hallein-Rif

Austria.

nils.haller@plus.ac.at

| Table 1. Study estimates (mean and 90% confidence limits in percent units) shown in the scatterplots of Figures 1 and 2. For more information about the studies see Part I (1). | | | | | | | | | | |  |
| --- | --- | --- | --- | --- | --- | --- | --- | --- | --- | --- | --- |
| **Study** | **Year** | **Training acronym** | **Extra details** | **Sprint speed/ power** | **Repeated-sprint ability** | **Time-trial speed/power** | **Peak speed/ power** | **Aerobic/ Anaerobic Threshold** | **V̇O_2max_** | **Exercise economy** | |
| **HIIT-group estimates for endurance athletes** | | | | | | | | | | |  |
| Clark et al. (2) | 2014 | Short HIT |  |  |  | **6.6;**  9.3 to 4.5 | **3.5;**  4.2 to 2.9 | **2.6;**  5.8 to -0.4 | **3.6;**  7.3 to 0.3 | **4.6;**  7.0 to 2.5 | |
|  |  | Long HIT |  |  |  | **4.2;**  6.3 to 2.3 | **7.3;**  11.7 to 3.6 | **3.7;**  6.0 to 1.6 | **2.2;**  5.2 to 0.7 | **3.7;**  6.5 to 1.1 | |
| Laursen et al. (3) | 2002 | G1 |  |  |  | **10.9;**  16.6 to 6.6 | **4.7;**  6.8 to 2.8 |  | **5.1;**  7.6 to 2.9 |  | |
|  |  | G2 |  |  |  | **11.5;**  15.8 to 8.8 | **5.9;**  7.8 to 4.3 |  | **7.7;**  10.0 to 6.0 |  | |
|  |  | G3 |  |  |  | **9.0;**  14.4 to 4.7 | **3.0;**  4.8 to 1.3 |  | **3.0;**  4.8 to 1.4 |  | |
| Rønnestad et al. (4) | 2019 | Exp |  |  |  |  | **4.9;**  7.6 to 2.4 | **3.8;**  5.9 to 2.0 | **3.7;**  5.5 to 2.1 | **0.4;**  6.0 to -4.9 | |
| Salazar-Martínez et al. (5) | 2018 | HIT |  |  |  | **2.2;**  4.2 to 0.3 |  |  | **2.0;**  5.1 to -0.9 |  | |
| Sandbakk et al. (6) | 2013 | SIG | 7 km |  |  | **4.6;**  6.7 to 2.8 |  | **9.4;**  14.9 to 5.0 | **4.4;**  5.7 to 3.3 |  | |
|  |  | SIG | 7 km |  |  | **6.6;**  10.0 to 3.8 |  | **9.4;**  14.9 to 5.0 | **4.4;**  5.7 to 3.3 |  | |
|  |  | LIG | 12 km |  |  | **1.5;**  3.2 to -0.1 |  | **1.6;**  6.3 to -2.8 | **2.9;**  5.4 to 0.6 |  | |
|  |  | LIG | 12 km |  |  | **0.9;**  3.4 to -1.5 |  | **1.6;**  6.3 to -2.8 | **2.9;**  5.4 to 0.6 |  | |
| Sandbakk et al. (7) | 2011 | IG |  |  |  | **4.6;**  7.1 to 2.4 |  | **9.4;**  13.3 to 6.4 | **3.9;**  5.6 to 2.5 |  | |
| Skovereng et al. (8) | 2018 | HIT |  |  |  | **6.5;**  8.7 to 4.8 | **3.2;**  4.9 to 1.6 |  | **6.4;**  7.2 to 5.9 | **-0.4;**  0.5 to -1.4 | |
| Smith et al. (9) | 2003 | HIT | 60% Tmax |  |  | **2.8;**  3.9 to 1.8 |  | **8.8;**  13.4 to 5.1 | **5.8;**  8.9 to 3.1 |  | |
|  |  | HIT | 60% Tmax |  |  | **2.3;**  4.7 to 0.1 |  | **8.8;**  13.4 to 5.1 | **5.8;**  8.9 to 3.1 |  | |
|  |  | HIT | 70% Tmax |  |  | **1.0;**  2.3 to -0.2 |  | **2.1;**  6.1 to -1.6 | **4.1;**  7.0 to 1.4 |  | |
|  |  | HIT | 70% Tmax |  |  | **0.3;**  2.3 to 1.6 |  | **2.1;**  6.1 to -1.6 | **4.1;**  7.0 to 1.4 |  | |
| Stepto et al. (10) | 1999 | HIT |  |  |  | **3.2;**  5.4 to 1.0 | **1.1;**  1.8 to 0.5 |  |  |  | |
| Stevens et al. (11) | 2015 | EBTSIT | 2 km |  |  | **4.3;**  7.8 to 1.1 |  | -**1.5;**  5.7 to -8.3 | **0.0;**  3.0 to -2.9 |  | |
|  |  | EBTSIT | 60 s |  |  | **2.9;**  3.8 to 2.0 |  | **-1.5;**  5.7 to -8.3 | **0.0;**  3.0 to -2.9 |  | |
| Stöggl et al. (12) | 2014 | HIT |  |  |  |  |  | **10.7;**  16.3 to 6.4 | **4.4;**  8.0 to 1.0 | **-3.1;**  4.5 to -10.1 | |
|  |  | POL |  |  |  |  |  | **6.4;**  7.9 to 5.4 | **11.0;**  18.1 to 5.5 | **-4.0;**  4.3 to -11.5 | |
|  |  | HVT |  |  |  |  |  | **9.4;**  16.9 to 3.2 | **9.8;**  14.4 to 6.4 | **-4.0;**  4.3 to -11.5 | |
| Sylta et al. (13) | 2016 | INC | 40 km |  |  | **2.4;**  4.1 to 0.8 | **5.2;**  7.1 to 3.6 | **0.8;**  3.4 to -1.7 | **5.6;**  7.6 to 3.9 | **-0.4;**  1.3 to -2.1 | |
|  |  | INC | 30 s |  |  | **6.6;**  9.5 to 4.2 | **5.2;**  7.1 to 3.6 | **0.8;**  3.4 to -1.7 | **5.6;**  7.6 to 3.9 | **-0.4;**  1.3 to -2.1 | |
|  |  | DEC | 40 km |  |  | **1.2;**  2.7 to -0.4 | **4.5;**  6.8 to 2.3 | **4.9;**  8.0 to 2.2 | **7.5;**  9.6 to 5.9 | **-0.5;**  1.5 to -2.4 | |
|  |  | DEC | 30 s |  |  | **7.9;**  11.0 to 5.4 | **4.5;**  6.8 to 2.3 | **4.9;**  8.0 to 2.2 | **7.5;**  9.6 to 5.9 | **-0.5;**  1.5 to -2.4 | |
|  |  | MIX | 40 km |  |  | **2.2;**  4.0 to 0.4 | **5.9;**  8.7 to 3.5 | **3.2;**  6.1 to 0.5 | **4.8;**  6.7 to 3.1 | **-0.2;**  1.5 to -1.9 | |
|  |  | MIX | 30 s |  |  | **3.4;**  5.3 to 1.7 | **5.9;**  8.7 to 3.5 | **3.2;**  6.1 to 0.5 | **4.8;**  6.7 to 3.1 | **-0.2;**  1.5 to -1.9 | |
| **Control-group estimates for endurance athletes** | | | | | | | | | | |  |
| Clark et al. (2) | 2014 |  |  |  |  | -**3.2;**  -0.3 to -5.9 | **-1.8;**  0.0 to -3.5 | **-3.5;**  0.4 to -7.1 | **-0.8;**  2.7 to -4.2 | 2.2;  4.9 to -0.4 | |
| Laursen et al. (3) | 2002 |  |  |  |  | -**2.1;**  0.2 to -4.3 | **-1.0;**  -0.1 to -1.8 |  | **0.8;**  2.6 to -0.9 |  | |
| Salazar-Martínez et al. (5) | 2018 |  |  |  |  | **1.0;**  5.6 to -4.3 |  |  | **-0.3;**  3.1 to -3.6 |  | |
| Sandbakk et al. (6) | 2013 |  |  |  |  | **1.0;**  2.4 to -0.3 |  | **1.2;**  5.9 to -3.2 | **0.0;**  2.8 to -2.7 |  | |
|  |  |  |  |  |  | **1.0;**  3.5 to -1.5 |  | **1.2;**  5.9 to -3.2 | **0.0;**  2.8 to -2.7 |  | |
| Sandbakk et al. (6) | 2011 |  |  |  |  | **-0.9;**  0.4 to -0.2 |  | **0.0;**  4.2 to -4.0 | **1.4;**  5.2 to -2.2 |  | |
| Smith et al. (9) | 2003 |  |  |  |  | **0.1;**  2.4 to -2.2 |  | **2.2;**  6.2 to -1.5 | **0.6;**  3.8 to -2.4 |  | |
|  |  |  |  |  |  | **-0.9;**  1.3 to -3.1 |  | **2.2;**  6.2 to -1.5 | **0.6;**  3.8 to -2.4 |  | |
| Soares-Caldeira et al. (14) | 2014 |  |  | **0.1;**  1.9 to -1.6 | **2.8;**  3.9 to 1.9 |  |  |  |  |  | |
| Stevens et al. (11) | 2015 |  |  |  |  | **0.4;**  2.2 to -1.4 |  | **1.6;**  9.1 to -5.4 | **-1.7;**  1.6 to -4.9 |  | |
|  |  |  |  |  |  | **1.2;**  1.8 to 0.5 |  | **1.6;**  9.1 to -5.4 | **-1.7;**  1.6 to -4.9 |  | |
| Stöggl et al. (12) | 2014 |  |  |  |  |  | **2.5;**  4.5 to 0.5 | **4.0;**  28.5 to -15.8 | **3.3;**  7.4 to -0.4 | **-0.1;**  18.7 to -16.0 | |
|  |  |  |  |  |  |  | **2.5;**  4.5 to 0.5 | **0.1;**  14.6 to -12.6 | **3.3;**  7.4 to -0.4 | -**0.1;**  18.7 to -16.0 | |
|  |  |  |  |  |  |  | **5.5;**  10.7 to 0.8 | **-1.5;**  15.1 to -15.6 | **2.0;**  7.7 to -3.4 | **-0.1;**  10.1 to -9.5 | |
|  |  |  |  |  |  |  | **5.5;**  10.7 to 0.8 | **1.8;**  8.7 to -4.6 | **2.0;**  7.7 to -3.4 | **-0.1;**  10.1 to -9.5 | |
| **HIIT-group estimates for non-endurance athletes** | | | | | | | | | | |  |
| Akdoğan et al. (15) | 2021 | SSG | 10 m | **-6.4;**  -4.5 to -7.9 | **-0.4;**  0.7 to -1.5 |  |  |  |  |  | |
|  |  | SSG | 30 m | **-3.5;**  -1.6 to -5.1 | **-0.4;**  0.7 to -1.5 |  |  |  |  |  | |
|  |  | SSG | 35 m | **-0.2;**  1.6 to -2.0 | **-0.4;**  0.7 to -1.5 |  |  |  |  |  | |
|  |  | SER | 10 m | **-4.1;**  -2.3 to -5.6 | **0.6;**  1.7 to -0.4 |  |  |  |  |  | |
|  |  | SER | 30 m | **-0.5;**  1.3 to -2.1 | **0.6;**  1.7 to -0.4 |  |  |  |  |  | |
|  |  | SER | 35 m | **0.8;**  2.5 to -0.9 | **0.6;**  1.7 to -0.4 |  |  |  |  |  | |
| Breil et al. (16) | 2010 | IT | VT1 |  |  |  |  | **2.1;**  7.6 to -3.1 | **5.9;**  7.9 to 4.2 |  | |
|  |  | IT | VT2 |  |  |  |  | **9.4;**  13.2 to 6.6 | **5.9;**  7.9 to 4.2 |  | |
| Helgerud et al. (17) | 2001 | HIT |  |  |  |  |  | **5.3;**  12.6 to -1.3 | **10.1;**  13.7 to 7.7 |  | |
| Hermassi et al. (18) | 2018 | Int |  | **2.9;**  4.4 to 1.5 | **2.7;**  3.7 to 1.8 |  |  |  |  |  | |
| Iaia et al. (19) | 2015 | SEP | 20 m | **1.4;**  3.0 to -0.2 | **-1.0;**  0.0 to -2.0 |  |  |  |  |  | |
|  |  | SEP | 40 m | **0.8;**  2.5 to -0.9 | **-1.0;**  0.0 to -2.0 |  |  |  |  |  | |
|  |  | SEP | 200 m | **2.2;**  4.3 to 0.1 | **-1.0;**  0.0 to -2.0 |  |  |  |  |  | |
|  |  | SEM | 20 m | **0.4;**  2.5 to -1.7 | **2.5;**  3.4 to 1.7 |  |  |  |  |  | |
|  |  | SEM | 40 m | **-0.4;**  1.1 to -1.8 | **2.5;**  3.4 to 1.7 |  |  |  |  |  | |
|  |  | SEM | 200 m | **1.2;**  1.7 to 0.7 | **2.5;**  3.4 to 1.7 |  |  |  |  |  | |
| Selmi et al. (20) | 2018 | RST-G |  | **4.3;**  6.2 to 2.7 | **5.4;**  7.6 to 3.5 |  |  |  |  |  | |
| Sheykhlouvand et al. (21) | 2018 | HIT |  |  |  |  |  | **4.8;**  9.3 to 0.7 | **7.3;**  11.9 to 3.3 |  | |
| Soares-Caldeira et al. (14) | 2014 | AddT |  | **1.4;**  2.7 to 0.1 | **2.5;**  5.3 to 1.6 |  |  |  |  |  | |
| Thomassen et al. (22) | 2010 | HI |  | **0.0;**  2.3 to -2.3 | **1.9;**  3.5 to 0.4 |  |  |  |  |  | |
| Yang et al. (23) | 2017 | HIT |  |  |  |  |  |  | **6.3;**  10.1 to 3.1 | **3.0;**  9.5 to -2.9 | |
| **Control-group estimates for non-endurance athletes** | | | | | | | | | | |  |
| Akdoğan et al. (15) | 2021 | CG | 10 m | **-5.6;**  -3.6 to -7.3 | **0.2;**  1.4 to -0.9 |  |  |  |  |  | |
|  |  | CG | 30 m | **-0.4;**  1.5 to -2.4 | **0.2;**  1.4 to -0.9 |  |  |  |  |  | |
|  |  | CG | 35 m | **0.4;**  2.4 to -1.5 | **0.2;**  1.4 to -0.9 |  |  |  |  |  | |
| Breil et al. (16) | 2010 |  |  |  |  |  |  | **0.7;**  8.2 to -6.2 | **2.8;**  6.3 to -0.5 |  | |
|  |  |  |  |  |  |  |  | **1.2;**  5.5 to -2.8 | **2.8;**  6.3 to -0.5 |  | |
| Helgerud et al. (17) | 2001 |  |  |  |  |  |  | **-1.7;**  4.5 to -7.6 | **1.9;**  4.9 to -1.0 |  | |
| Hermassi et al. (18) | 2018 |  |  | **0.6;**  2.1 to -0.8 | **-0.3;**  0.7 to -1.2 |  |  |  |  |  | |
| Selmi et al. (20) | 2018 |  |  | **-0.8;**  0.4 to -1.9 | **-1.0;**  0.2 to -2.1 |  |  |  |  |  | |
| Sheykhlouvand et al. (21) | 2018 |  |  |  |  |  |  | **1.0;**  5.2 to -3.1 | **1.5;**  3.1 to 0.0 |  | |
|  |  |  |  |  |  |  |  | **1.0;**  5.2 to -3.1 | **1.5;**  3.1 to 0.0 |  | |
| Thomassen et al. (22) | 2010 |  |  | **0.0;**  1.7 to -1.7 | **-2.1;**  -1.2 to -2.9 |  |  |  |  |  | |
| Wells et al. (24) | 2014 |  |  |  |  |  | **-2.1;**  -1.0 to -3.2 |  | **0.9;**  5.6 to -3.7 |  | |
| ^a^ High-intensity interval training  ^b^ Distance or time for sprints or time trials; type of aerobic/anaerobic threshold. | | | | | | | | | | |  |

**References**

1. Wiesinger H-P, Stöggl T, Haller N, Blumkaitis J, Strepp T, Kilzer F, et al. Meta-analyses of the effects of high-intensity interval training in elite athletes – part I: mean effects on various performance measures. Front Physiol. 2024;accepted.

2. Clark B, Costa VP, O'Brien BJ, Guglielmo LG, Paton CD. Effects of a seven day overload-period of high-intensity training on performance and physiology of competitive cyclists. PLoS One. 2014;9(12):e115308.

3. Laursen PB, Shing CM, Peake JM, Coombes JS, Jenkins DG. Interval training program optimization in highly trained endurance cyclists. Med Sci Sports Exerc. 2002;34(11):1801-7.

4. Rønnestad BR, Vikmoen O. A 11-day compressed overload and taper induces larger physiological improvements than a normal taper in elite cyclists. Scand J Med Sci Sports. 2019;29(12):1856-65.

5. Salazar-Martinez E, Santalla A, Orellana JN, Strobl J, Burtscher M, Menz V. Influence of high-intensity interval training on ventilatory efficiency in trained athletes. Respir Physiol Neurobiol. 2018;250:19-23.

6. Sandbakk Ø, Sandbakk SB, Ettema G, Welde B. Effects of intensity and duration in aerobic high-intensity interval training in highly trained junior cross-country skiers. J Strength Cond Res. 2013;27(7):1974-80.

7. Sandbakk Ø, Welde B, Holmberg HC. Endurance training and sprint performance in elite junior cross-country skiers. J Strength Cond Res. 2011;25(5):1299-305.

8. Skovereng K, Sylta Ø, Tønnessen E, Hammarström D, Danielsen J, Seiler S, et al. Effects of initial performance, gross efficiency and V˙O2peak characteristics on subsequent adaptations to endurance training in competitive cyclists. Front Physiol. 2018;9:713.

9. Smith TP, Coombes JS, Geraghty DP. Optimising high-intensity treadmill training using the running speed at maximal O(2) uptake and the time for which this can be maintained. Eur J Appl Physiol. 2003;89(3-4):337-43.

10. Stepto NK, Hawley JA, Dennis SC, Hopkins WG. Effects of different interval-training programs on cycling time-trial performance. Med Sci Sports Exerc. 1999;31(5):736-41.

11. Stevens AWJ, Olver TT, Lemon PWR. Incorporating sprint training with endurance training improves anaerobic capacity and 2,000-m erg performance in trained oarsmen. J Strength Cond Res. 2015;29(1):22-8.

12. Stöggl T, Sperlich B. Polarized training has greater impact on key endurance variables than threshold, high intensity, or high volume training. Front Physiol. 2014;5:33.

13. Sylta O, Tonnessen E, Hammarstrom D, Danielsen J, Skovereng K, Ravn T, et al. The effect of different high-intensity periodization models on endurance adaptations. Med Sci Sports Exerc. 2016;48(11):2165-74.

14. Soares-Caldeira LF, de Souza EA, de Freitas VH, de Moraes SM, Leicht AS, Nakamura FY. Effects of additional repeated sprint training during preseason on performance, heart rate variability, and stress symptoms in futsal players: a randomized controlled trial. J Strength Cond Res. 2014;28(10):2815-26.

15. Akdoğan E, Yılmaz İ, Köklü Y, Alemdaroğlu U, Cerrah AO. The effect of isolated or combined small-sided games and speed endurance training on physical performance parameters in young soccer players. Kinesiology. 2021;53(1):78-85.

16. Breil FA, Weber SN, Koller S, Hoppeler H, Vogt M. Block training periodization in alpine skiing: effects of 11-day HIT on VO2max and performance. Eur J Appl Physiol. 2010;109(6):1077-86.

17. Helgerud J, Engen LC, Wisløff U, Hoff J. Aerobic endurance training improves soccer performance. Med Sci Sports Exerc. 2001;33(11):1925-31.

18. Hermassi S, Ingebrigtsen J, Schwesig R, Fieseler G, Delank KS, Chamari K, et al. Effects of in-season short-term aerobic and high-intensity interval training program on repeated sprint ability and jump performance in handball players. J Sports Med Phys Fitness. 2018;58(1):50-6.

19. Iaia FM, Fiorenza M, Perri E, Alberti G, Millet GP, Bangsbo J. The effect of two speed endurance training regimes on performance of soccer players. PLoS ONE. 2015;10(9):e0138096.

20. Selmi W, Rebai H, Chtara M, Naceur A, Sahli S. Self-confidence and affect responses to short-term sprint interval training. Physiol Behav. 2018;188:42-7.

21. Sheykhlouvand M, Gharaat M, Khalili M, Agha-Alinejad H, Rahmaninia F, Arazi H. Low-volume high-intensity interval versus continuous endurance training: effects on hematological and cardiorespiratory system adaptations in professional canoe polo athletes. J Strength Cond Res. 2018;37(7):1852-60.

22. Thomassen M, Christensen PM, Gunnarsson TP, Nybo L, Bangsbo J. Effect of 2-wk intensified training and inactivity on muscle Na+-K+ pump expression, phospholemman (FXYD1) phosphorylation, and performance in soccer players. J Appl Physiol (1985). 2010;108(4):898-905.

23. Yang MT, Lee MM, Hsu SC, Chan KH. Effects of high-intensity interval training on canoeing performance. Eur J Sport Sci. 2017;17(7):814-20.

24. Wells C, Edwards A, Fysh M, Drust B. Effects of high-intensity running training on soccer-specific fitness in professional male players. Appl Physiol Nutr Metab. 2014;39(7):763-9.
